# RandMScan: accelerating parallel scan via matrix computation and random-jump strategy

**DOI:** 10.1038/s41598-025-32283-5

**Published:** 2025-12-24

**Authors:** Shujun Peng, Xinhan Lin, Yu Zhang, Yuheng Xiao, Yang Hu

**Affiliations:** 1 Shanghai Artificial Intelligence Laboratory, Shanghai, 200232 China; 2 Tsinghua University, Beijing, 100084 China

**Keywords:** Engineering, Mathematics and computing

## Abstract

*Parallel scan* is a fundamental primitive widely used in a broad range of workloads, including parallel sorting, graph algorithms, and sampling in large language model inference. Although GPU-optimized parallel scan algorithms have been extensively studied, their reliance on vector units makes them inefficient on modern AI accelerators, which typically lack such units but incorporate abundant processing element (PE) arrays tailored for matrix operations. Existing matrix-based approaches, however, suffer from excessive bandwidth consumption due to auxiliary matrix loading as well as costly inter-block communication, thereby limiting their scalability in practical deployments. In this paper, we propose RandMScan, a two-stage parallel scan framework specifically designed for AI accelerators equipped with PE arrays, to address the aforementioned challenges. The first stage employs an efficient matrix-based local chunk scan algorithm that fully exploits fine-grained parallelism within PE arrays, while the second stage introduces a lightweight *Random-Jump* strategy to coordinate global aggregation with reduced synchronization overhead. Together, these techniques enable scalable execution over long sequences while effectively mitigating the substantial communication overhead that typically arises in prior solutions. Extensive evaluations on state-of-the-art AI accelerators demonstrate that our method achieves more than 80% speedup compared to existing matrix-based implementations for long input sequences, and can reduce the end-to-end latency by 15%–26% in representative downstream applications of scan.

## Introduction

Scan, or prefix sum, computes cumulative reductions over a sequence, producing each output as the sum of all preceding elements. Given an input list ${x_1, x_2, \ldots , x_{n}}$, the inclusive scan yields ${y_1, y_2, \ldots , y_{n}}$ with $y_i = x_1 + x_2 + \cdots + x_i$. For example, applying it to [4, 1, 7, 0, 3] produces [4, 5, 12, 12, 15]. In contrast, the exclusive scan outputs [0, 4, 5, 12, 12], omitting the current element from its own sum. As a fundamental primitive, scan is widely used in many computational tasks. It plays a central role in parallel radix sorting^1,2^, weighted sampling^3^, and more recently, in next-token prediction for large language models (LLMs)^4,5^.

Despite its broad applicability, scan can become a significant performance bottleneck in practice. As shown in Table 1, our profiling on an NVIDIA A800 GPU using NVIDIA Nsight Systems and Nsight Compute indicates that scans account for 22%–25% of execution time in parallel radix sort and 26%–39% in top-*p* sampling, highlighting the importance of optimizing this primitive for overall performance.

Although scan is trivial to implement sequentially, the inherent recursive data dependencies between elements make its parallelization challenging. Consequently, significant research efforts have been devoted to developing efficient parallel scan algorithms.

The implementation of scan operations on classical parallel architectures like GPUs has been extensively studied. For long input sequences, a two-stage approach is commonly adopted: the *local stage* computes independent scan results within each block or chunk, while the *global stage* aggregates these partial results to produce the complete scan output. Commonly used algorithms in the local stage include classic parallel prefix trees^6–8^ and their improved variants designed for different performance trade-offs. These structures are conveniently implemented on vector-parallel processing units such as GPU CUDA cores and are integrated into widely used libraries like CUB^9^. Approaches to the global stage can be broadly categorized into hierarchical methods^10^ that require multiple rounds of reads and writes, and inter-block communication schemes^11,12^ that leverage global memory for synchronization.

**Table 1:** Scan time as a percentage of total execution time for radix sort and top-$p$ sampling across various input lengths.

Sequence length	Radix sort (%)	Top-*p* sampling (%)
16k	22.27	19.72
32k	23.74	26.59
64k	25.43	33.14
128k	24.18	35.78
256k	23.93	39.08

Modern GPUs and many deep learning accelerators are increasingly equipped with dedicated matrix computation units, such as NVIDIA’s Tensor Cores^13^ and the Cube Units in Huawei’s Ascend series^14^. This has inspired efforts to reformulate scan as a matrix multiplication problem, enabling efficient execution on these units^15,16^. The key idea is to first reshape the input sequence into a matrix, then perform matrix operations with specially structured matrices–such as upper-triangular matrices or all-ones matrix–and finally flatten the result to obtain the scanned sequence. Recently, Wroblewski et al.^5^ build upon the algorithm proposed by Dakkak et al.^15^ and implement an optimized version on Huawei’s Ascend accelerator, leveraging its Cube Units to accelerate the local stage scan computation.

However, the aforementioned methods face several significant challenges when deployed on resource-constrained edge AI accelerators. These accelerators often sacrifice vector processing units to increase the area of dedicated processing units and PE arrays for matrix-related processing^17,18^, making conventional local stage scan algorithms^6–9^ particularly inefficient, as their vector execution patterns must be emulated through suboptimal PE array utilization. While some matrix-based strategies, such as the algorithm from Dakkak et al.^15^, Algorithm 6 and the ScanUL algorithm from Wroblewski et al.,^5^ remain executable under these constraints, they require loading multiple auxiliary matrices, leading to significant data movement overhead on bandwidth-limited accelerators and further increasing latency. At the global stage, the decoupled look-back strategy^11^ relies heavily on fine-grained SIMT parallelism capabilities found in GPUs, making it ill-suited for platforms lacking such features. Moreover, due to the lack of a global barrier on edge AI accelerators, the global scan method from Wroblewski et al.^5^ degrades to the hierarchical approach^10^, which incurs high memory overhead because of repeated reads and writes of intermediate results.

In this paper, we propose RandMScan, a parallel scan implementation method designed specifically for efficient execution on AI accelerators, which also operates in two stages: the local chunk stage and the global stage. At the local chunk stage, we introduce an intra-chunk collaborative computation algorithm that relies solely on matrix computations to collaboratively process multiple blocks within each chunk, enabling highly efficient execution by processing elements (PEs). This collaborative design not only enhances computational resource utilization but also reduces the number of auxiliary matrices required by the methods of Wroblewski et al.^5^ and Dakkak et al.^15^ from three to two, significantly lowering memory traffic overhead. At the global stage, we propose a Random-Jump communication strategy that transforms local scan results into the final global scan output. This approach avoids the multiple rounds of data reads present in the approaches of Wroblewski et al.^5^ and Dakkak et al.^15^ and eliminates the need for SIMT units for communication^11^.

The effectiveness of our Random-Jump strategy is validated on GPUs through a fair comparison, where the local stage implementation is fixed to ensure consistency. On state-of-the-art accelerators^17^ equipped with PE arrays, implementation results show that our approach achieves 73%−87% performance improvement compared to existing scan methods^5,11,15^. Furthermore, RandMScan can reduce execution time by up to 26% on several representative downstream tasks that rely on the scan operation, highlighting the practical effectiveness and broad applicability of our method.

## Background and related work

### Parallel hardware and their applications

GPUs adopt a massively parallel architecture with thousands of lightweight CUDA cores organized into streaming multiprocessors (SMs), making them well-suited for data-parallel workloads such as vector operations and reductions. Parallel Scan on such conventional GPU cores have been extensively studied over the past decades^9–12^, with many efficient implementations tailored to the vector-based execution mode. While these vector-based methods are highly optimized for conventional GPU architectures, they do not directly translate to the emerging matrix engines.

Recent years have witnessed the proliferation of heterogeneous processors that integrate specialized matrix engines–such as Google TPUs^19^, NVIDIA Tensor Cores^20^, AMD Matrix Cores^21^, and Huawei Ascend Cube Units^14^–designed to accelerate dense linear algebra. These units can execute a large number of fused multiply–add operations on small matrices per instruction, offering massive fine-grained parallelism and superior energy efficiency compared to traditional vector pipelines. Nevertheless, many parallel algorithms originally designed for vector units can become inefficient when directly ported to such units designed for matrix computation, since their execution model and memory access patterns are fundamentally different. To formalize this computational model, Chowdhury et al.^16^ introduced the Tensor Core Unit (TCU) abstraction, which enables classical algorithms to be reformulated in terms of matrix operations. In a similar vein, Navarro et al.^22^, Carrasco et al.^23^, and Dakkak et al.^15^ demonstrated that reduction and scan primitives can be re-expressed as a sequence of small-matrix multiplications and thus efficiently accelerated by TCUs. In addition, other non-matrix multiplicative tasks, such as k-nearest neighbor (KNN) search^24^, DBSCAN^25^ and Euclidean distance computation^26^, have also been effectively mapped onto tensor cores to exploit their high-throughput matrix processing capabilities.

The recent surge of AI applications has also driven the development of a wide range of domain-specific accelerators^17,27,28^. In order to maximize efficiency, these accelerators often dedicate specialized hardware units to common AI kernels such as Attention^29^ and Convolution^30^, rather than relying on generic tensor cores as in modern GPUs. To retain flexibility, many of them also integrate relatively large processing element (PE) arrays that can be reconfigured to support diverse workloads. For instance, Wang et al.^17^ present SOFA, which incorporates two $128\times 128$PE arrays, while Qin et al.^27^ propose FACT, which adopts a $16\times 32$ PE array. Such PE arrays can be partitioned into multiple fine-grained matrix computation units or operated as a single large unit, thereby balancing programmability with high utilization of the underlying hardware resources.

### Related work on parallel scan

**Fig. 1:**
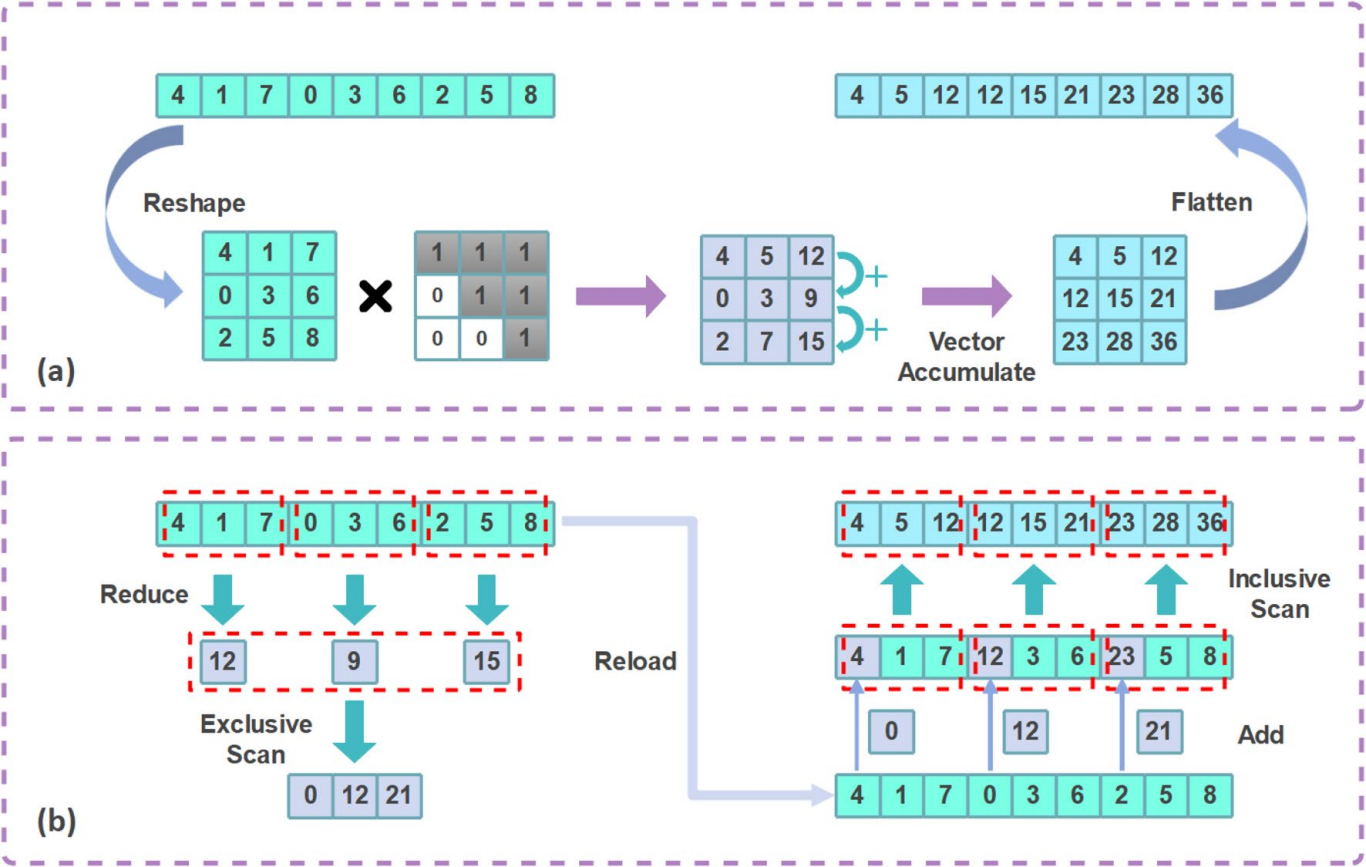
(**a**) Simplified ScanU algorithm from Wroblewski et al.^5^ (**b**) Simplified RSS strategy.

At the *local stage*, classical vectorized scan algorithms–such as parallel prefix trees including Kogge–Stone^6^, Sklansky^8^, Brent–Kung^7^, along with their numerous variants^9^–dominate GPU implementations. An alternative is the matrix-based approach, as shown in Fig. 1(a), where a simplified version of the ScanU algorithm from Wroblewski et al.^5^ reshapes the input sequence into a matrix, right-multiplies it by an upper-triangular matrix, and then uses vector units to perform sequential accumulation on the intermediate results, yielding the scan result. A purely matrix-based alternative is the method of Dakkak et al.,^15^ which reformulates scan as:

1 $$\begin{aligned} \textrm{scan}(\textbf{z}) = \textbf{A} \cdot \textbf{U} + \textbf{L}^{-} \cdot (\textbf{A} \cdot \textbf{1}), \end{aligned}$$

where $\textbf{A} \in \mathbb {R}^{s \times s}$ is reshaped from the input sequence $\textbf{z}$, $\textbf{U} \in \mathbb {R}^{s \times s}$ and $\textbf{L}^{-} \in \mathbb {R}^{s \times s}$ are upper and strictly lower triangular matrices respectively, and $\textbf{1} \in \mathbb {R}^{s \times 1}$ is an all-ones vector. The resulting matrix $\textrm{scan}(\textbf{z}) \in \mathbb {R}^{s \times s}$ is then flattened back into a one-dimensional sequence to yield the final scan output. Such a matrix-only approach is naturally more suitable for hardware equipped with PE arrays. However, loading the three special auxiliary matrices $\textbf{U}$, $\textbf{L}^{-}$, and $\textbf{1}$ introduces additional memory footprint, which can lead to high latency on bandwidth-limited accelerators.

At the *global stage*, where partial results from local scans must be aggregated into global scan results, widely adopted hierarchical strategies–such as Scan-Scan-Add (SSA) and Reduce-Scan-Scan (RSS)–organize the scan computation into multiple passes, incurring substantial memory traffic overhead: approximately 4 N and 3 N memory accesses respectively^10^. As illustrated in Fig. 1(b), a simplified RSS variant first performs an independent reduction within each block to obtain partial sums. These partial sums are then subjected to an exclusive scan to generate global prefix offsets, which are subsequently added to the first element of each corresponding block. Finally, an inclusive scan is performed within each block to produce the complete scan result. This hierarchical structure is also used in the matrix-based scan method of Dakkak et al.^15^ and custom hardware modules for scan-based applications^2^, resulting in similar high memory traffic overhead.

In contrast, the StreamScan algorithm by Yan et al.^12^ reduces the memory traffic to the theoretical minimum of 2 N (N reads and N writes), but introduces inter-block dependencies that necessitate synchronization between adjacent thread blocks on GPUs. To mitigate the resulting synchronization latency, Merrill et al.^11^ propose a decoupled look-back strategy that efficiently coordinates inter-block communication through warp-level GPU primitives. Recently, the global scan method proposed by Wroblewski et al.^5^ achieved optimal performance on Ascend accelerators. However, on platforms lacking hardware-supported global barriers, it reverts to two-pass data movement schemes like those proposed by Dakkak et al.^15^ and Harris et al.,^10^ incurring significant additional latency.

## Proposed method

This section presents the detailed design of RandMScan, which consists of two sequential stages. To efficiently process long input sequences, the data is first divided into multiple chunks, with each chunk undergoing a local scan result computation in the *local chunk stage*. The partial scan results within each chunk are then aggregated in the *global stage*, where global communication and cumulative addition across all preceding chunks are performed to produce the correct scan results for the entire sequence.

### Local chunk stage

**Fig. 2:**
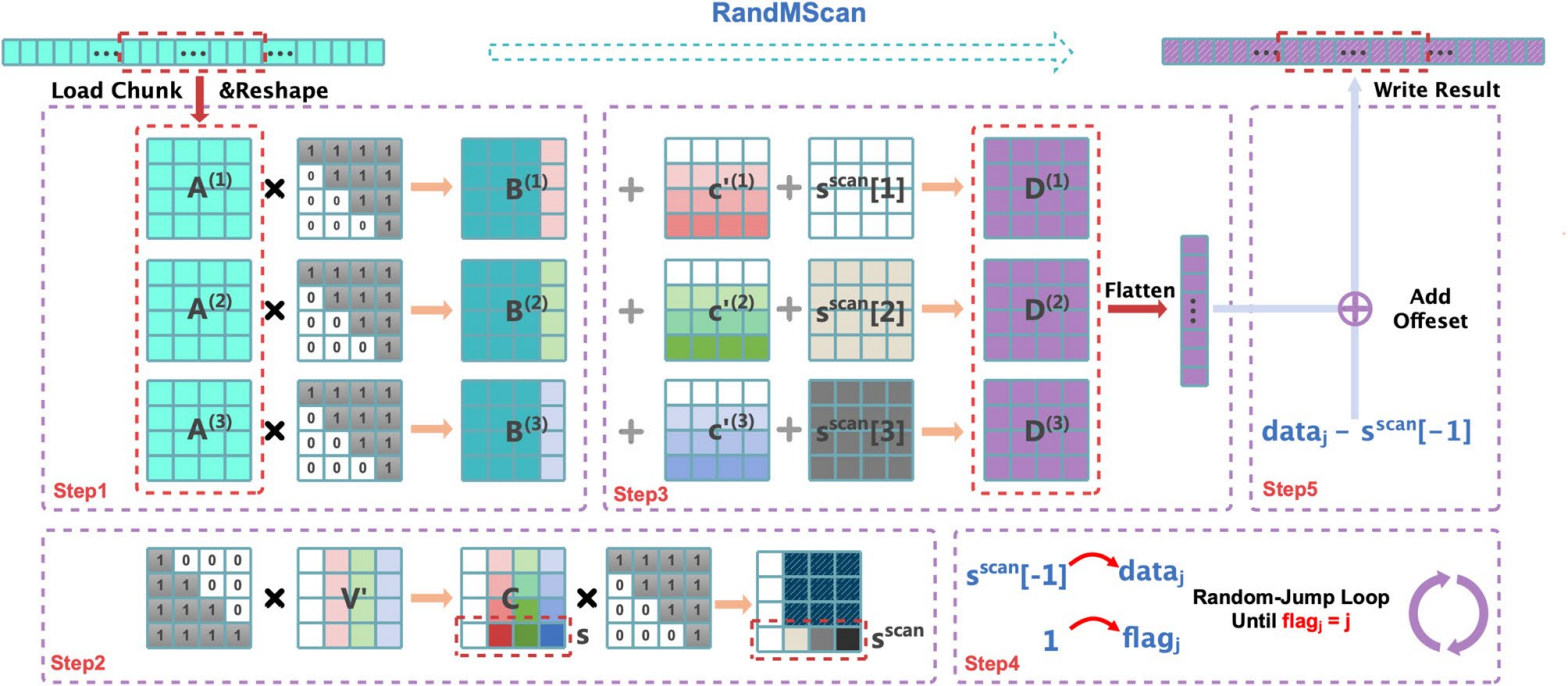
The workflow of RandMScan for $k = 4$. Steps 1–3 correspond to the local chunk scan stage, while Steps 4 and 5 belong to the global stage.

In the local chunk stage of RandMScan, we introduce an intra-chunk collaborative computation algorithm designed to efficiently compute the local scan results within each chunk. For long sequences, the original input is partitioned into multiple chunks, with each chunk containing a data segment *x* of length $l = (k{-}1)k^2$, where the parameter *k* is determined by the underlying matrix computation granularity of the PE arrays in accelerator. For instance, a $128 \times 128$ PE array can be flexibly configured as $16 \times 16$ processing units, each capable of executing independent matrix operations on $8 \times 8$ sub-matrices, corresponding to $k = 8$. Alternatively, it can be configured as $32 \times 32$ processing units, each handling $4 \times 4$ sub-matrices, corresponding to $k = 4$. This chunk formulation ensures that the input size is fully compatible with the computational structure of the PE array. In the case where the original chunk length does not match the required size, zero-padding is applied to the end of the chunk to guarantee proper alignment with the matrix-based operations. Figure 2 illustrates the complete processing flow of RandMScan for the representative case of $k = 4$, where Steps 1–3 correspond to the local chunk scan stage, and Steps 4–5 will be further explained as part of the *Random-Jump* strategy in the subsequent section.

**Algorithm 1:**
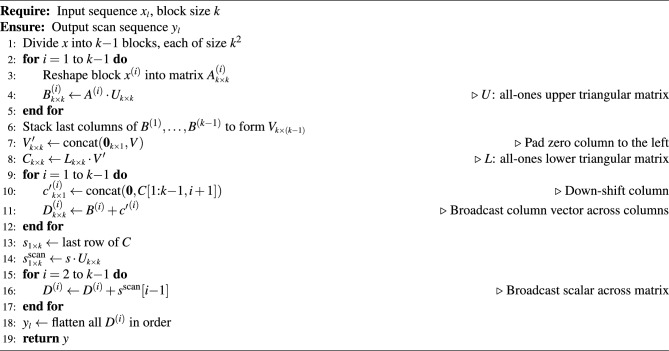
Matrix-based Local Chunk Stage Algorithm

A detailed step-by-step description of our local-stage algorithm is presented in Algorithm 1. Specifically, the data within each chunk is divided into $k{-}1$ consecutive blocks of size $k^2$. To execute the procedure efficiently on the PE array, we employ $k$ matrix processing units in a collaborative manner. Each block is reshaped into a square matrix $A^{(i)}_{\scriptscriptstyle k \times k}$, which is then assigned to one of the $k{-}1$ processing units. An additional auxiliary unit is employed to aggregate partial results across blocks and to produce the final chunk-level output. For each block, the computation proceeds as follows. We first perform a matrix multiplication with an all-ones upper triangular matrix *U*, yielding $B^{(i)} = A^{(i)} \cdot U$. This step encodes the row-wise prefix sums within each block matrix, thereby capturing the intra-row cumulative structure. From each resulting matrix $B^{(i)}$, the last column is extracted, and these columns are stacked together to form the matrix $V_{\scriptscriptstyle k \times (k{-}1)}$. To enable uniform processing, we prepend a zero column to $V$, resulting in $V'_{\scriptscriptstyle k \times k}$. We then compute the matrix product $C = L \cdot V'$, where *L* is an all-ones lower triangular matrix. The matrix *C* effectively encodes the cumulative row sums across all blocks within the chunk, serving as the intermediate state required to propagate partial sums between blocks.

The next step ensures that the cumulative results encoded in *C* are correctly propagated back to each block. Specifically, for each block index *i*, we down-shift the $i+1$-th column of *C*, pad it with zero at the top to get ${c'}^{(i)}$, and broadcast the resulting column vector across all columns of $B^{(i)}$. This yields the updated block $D^{(i)} = B^{(i)} + {c'}^{(i)}$, which represents the intra-block scan result of $A^{(i)}$.

To finalize the intra-chunk(i.e. inter-block) scan, we extract the last row of *C*, denoted as $s_{\scriptscriptstyle 1 \times k}$, and compute its prefix sums through multiplication with the upper triangular matrix *U*, yielding $s^{\text {scan}} = s \cdot U$. These scalar values are then broadcasted to all elements within the corresponding matrices $D^{(i)}$, thereby ensuring consistency across block boundaries. Finally, the full scan result for the chunk, *y*, is obtained by flattening all matrices $D^{(i)}$ in their original order.

Overall, the complete local chunk scan over $k{-}1$ blocks requires only $k{+}1$ matrix multiplications and $2k{-}2$ matrix additions. Importantly, the method relies on just two special matrices–an upper triangular matrix *U* and a lower triangular matrix *L*. Each of the $k{-}1$ parallel processing units is only required to access the upper triangular matrix *U*, thereby minimizing memory bandwidth consumption. In contrast, the conventional matrix-based method^5,15^ expressed in Equation 1 requires 3 matrix multiplications, 1 matrix addition, and the loading of 3 distinct special matrices for processing a *single* block. This highlights that our approach not only achieves superior computational efficiency but also substantially reduces memory access overhead, making it particularly well-suited for execution on modern AI accelerators with PE arrays.

**Algorithm 2:**
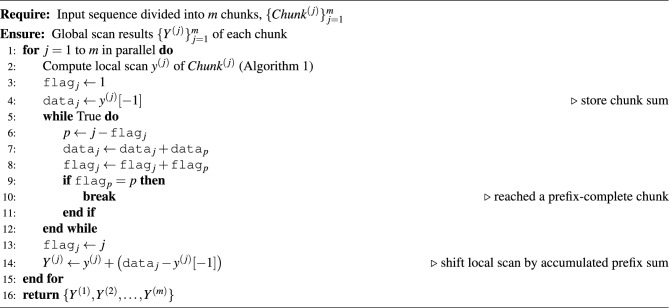
Global Scan Algorithm Using Random Jump Strategy

### Global stage: random-jump strategy

After performing the local stage scan within each chunk, it becomes necessary to determine the sum of all preceding segments and add it to local chunk scan result to obtain the correct global scan result. In RandMScan, we propose a Random-Jump communication mechanism that efficiently propagates prefix segments sums across chunks. This mechanism avoids excessive memory traffic and synchronization overhead, ensuring high performance and scalability. Specifically, for each chunk, we allocate a flag and a data value in global memory or on-chip shared SRAM memory if available. The flag indicates the readiness and range of data, while the data holds the partial accumulated sum. A flag value of 0 means that the result for the current chunk has not yet been computed, whereas a value of 1 means that only the local (intra-chunk) sum is available. If the flag value of the *i*-th chunk is *k* (where $k> 1$), it indicates that the corresponding data value stores the accumulated sum from chunk $i{-}k{+}1$ to *i*, i.e., $\sum _{j=i-k+1}^{i} x_j$.

AS shown in Algorithm 2, during the scan process, for the *j*-th chunk, we first compute its intra-chunk scan. Once completed, we set the $\texttt {flag}_j$ to 1 and write the chunk’s sum (i.e., $s^{\text {scan}}[-1]$, the last element of its scan result) to the $\texttt {data}_j$ field. Then, each time, we will read the status of the ($j-\texttt {flag}_j$)-th chunk, add $\texttt {data}_{j-\texttt {flag}_j}$ to $\texttt {data}_{j}$, $\texttt {flag}_{j-\texttt {flag}_j}$ to $\texttt {flag}_{j}$, respectively. This loop continues until we encounter a preceding chunk $\alpha$ whose flag equals $\alpha$ (i.e., $\texttt {flag}_\alpha = \alpha$). At that point, the full prefix sum up to chunk *j* can be determined by $\texttt {data}_j+\texttt {data}_\alpha$. We then update the data of chunk *j* to store this total sum and set $\texttt {flag}_j$ to *j*, indicating that $\texttt {data}_j$ is the cumulative sum of chunks 1 to *j*. Finally, we add $\texttt {data}_j - s^{\text {scan}}[-1]$ to the current chunk’s local scan results, thereby completing the global stage scan for this chunk.

**Table 2:** Detailed process of the Random-Jump strategy with eight chunks. Each round contains a *jump* phase (showing the jump targets) and an *update* phase (showing the resulting (flag, data)).

Round	Phase	$\mathbf {C_1}$	$\mathbf {C_2}$	$\mathbf {C_3}$	$\mathbf {C_4}$	$\mathbf {C_5}$	$\mathbf {C_6}$	$\mathbf {C_7}$	$\mathbf {C_8}$
**0 (Init)**	Jump	/	/	/	/	/	/	/	/
	Update	(1, 7)	(1, 15)	(1, 9)	(1, 20)	(1, 5)	(1, 14)	(1, 11)	(1, 8)
**1**	Jump	/	$\rightarrow C_1$	$\rightarrow C_2$	$\rightarrow C_3$	$\rightarrow C_4$	**Stall**	$\rightarrow C_6$	$\rightarrow C_7$
	Update	(1, 7)	(2, 22)	(2, 24)	(2, 29)	(2, 25)	(1, 14)	(2, 25)	(2, 19)
**2**	Jump	/	/	$\rightarrow C_1$	$\rightarrow C_2$	$\rightarrow C_3$	$\rightarrow C_5$	$\rightarrow C_5$	$\rightarrow C_6$
	Update	(1, 7)	(2, 22)	(3, 31)	(4, 51)	(4, 49)	(3, 43)	(4, 50)	(4, 38)
**3**	Jump	/	/	/	/	$\rightarrow C_1$	$\rightarrow C_3$	$\rightarrow C_3$	$\rightarrow C_4$
	Update	(1, 7)	(2, 22)	(3, 31)	(4, 51)	(5, 56)	(6, 70)	(7, 81)	(8, 89)
**4 (Final)**	Jump	/	/	/	/	/	/	/	/
	Update	(1, 7)	(2, 22)	(3, 31)	(4, 51)	(5, 56)	(6, 70)	(7, 81)	(8, 89)

To provide an intuitive illustration of the principle behind the Random-Jump strategy, Table 2 presents a concrete example with eight chunks. The local scan results of all chunks form the sequence:

$$X = [7,\ 15,\ 9,\ 20,\ 5,\ 14,\ 11,\ 8].$$

Each chunk $C_j$ is initialized with $(\texttt {flag}_j = 1,\ \texttt {data}_j = X_j)$, where $\texttt {flag}=1$ indicates that only the intra-chunk scan has been completed.

Then, in each round, chunks perform their jump operations according to Algorithm 2. In Round 1, all chunks except the first one perform their first jump toward the preceding chunk, e.g., $C_2 \rightarrow C_1$, $C_3 \rightarrow C_2$, and so on. To demonstrate the robustness of the algorithm, the sixth chunk $C_6$ is intentionally stalled in this round (indicated as “**Stall**” in Table 2). Despite this delay, the subsequent rounds correctly converge to the final global prefix results, showing that the Random-Jump mechanism can tolerate asynchronous progress and transient stalls without affecting correctness.

After all Random-Jump iterations, the final $\texttt {data}_j$ values converge to the global scan result:

$$[\texttt {data}_1,\ \texttt {data}_2,\ \ldots ,\ \texttt {data}_8] = [7,\ 22,\ 31,\ 51,\ 56,\ 70,\ 81,\ 89],$$

which exactly corresponds to the scan result of *X*.

It is worth noting that, in hardware, Random-Jump operations do not require global synchronization across all chunks. Each chunk can proceed as soon as its required predecessor data become available, enabling highly parallel and fine-grained execution. In this example, we synchronize all chunks per round only for the sake of clarity in visualization and to better demonstrate convergence behavior under a controlled stall scenario. Unlike the method of Wroblewski et al.,^5^ our strategy only requires each chunk to communicate and synchronize with two data items, and does not require a global barrier, making it particularly suitable for edge accelerators with simple control logic.

## Experiments

In this section, we present a thorough evaluation of the proposed RandMScan method, focusing on the effectiveness of the Random-Jump strategy in the global stage as well as the overall performance of the complete scan algorithm across a variety of length and downstream tasks.

To rigorously validate the performance of the Random-Jump mechanism, we conduct a comparative study against several established global-stage scan methods^5,9,11,15^ using an NVIDIA A800 GPU as the test platform. For fairness, all approaches employ the CUB library’s BlockScaninterface to perform the local stage computation. In addition, we implement both single-threaded (ST) and multi-threaded (MT) variants of RandMScan and the Decoupled method proposed by Merrill et al.^11^. The single-threaded versions are designed to emulate edge AI accelerators that lack SIMT execution units for inter-chunk communication, thereby providing insight into the performance of our approach under constrained hardware conditions.

During these experiments, we verified the correctness and efficiency of the Random-Jump strategy using both fp32 and int32 data types. Since the A800 GPU provides equivalent throughput for these types, no noticeable latency difference was observed. As expected, all floating-point implementations exhibit minor numerical deviations due to the non-associativity of floating-point addition; in particular, pairwise comparisons among our method and the approaches of^5,11,15^ show relative deviations on the order of $10^{-7}$ to $10^{-6}$, which are fully acceptable for floating-point applications such as top-*p* sampling.

**Fig. 3:**
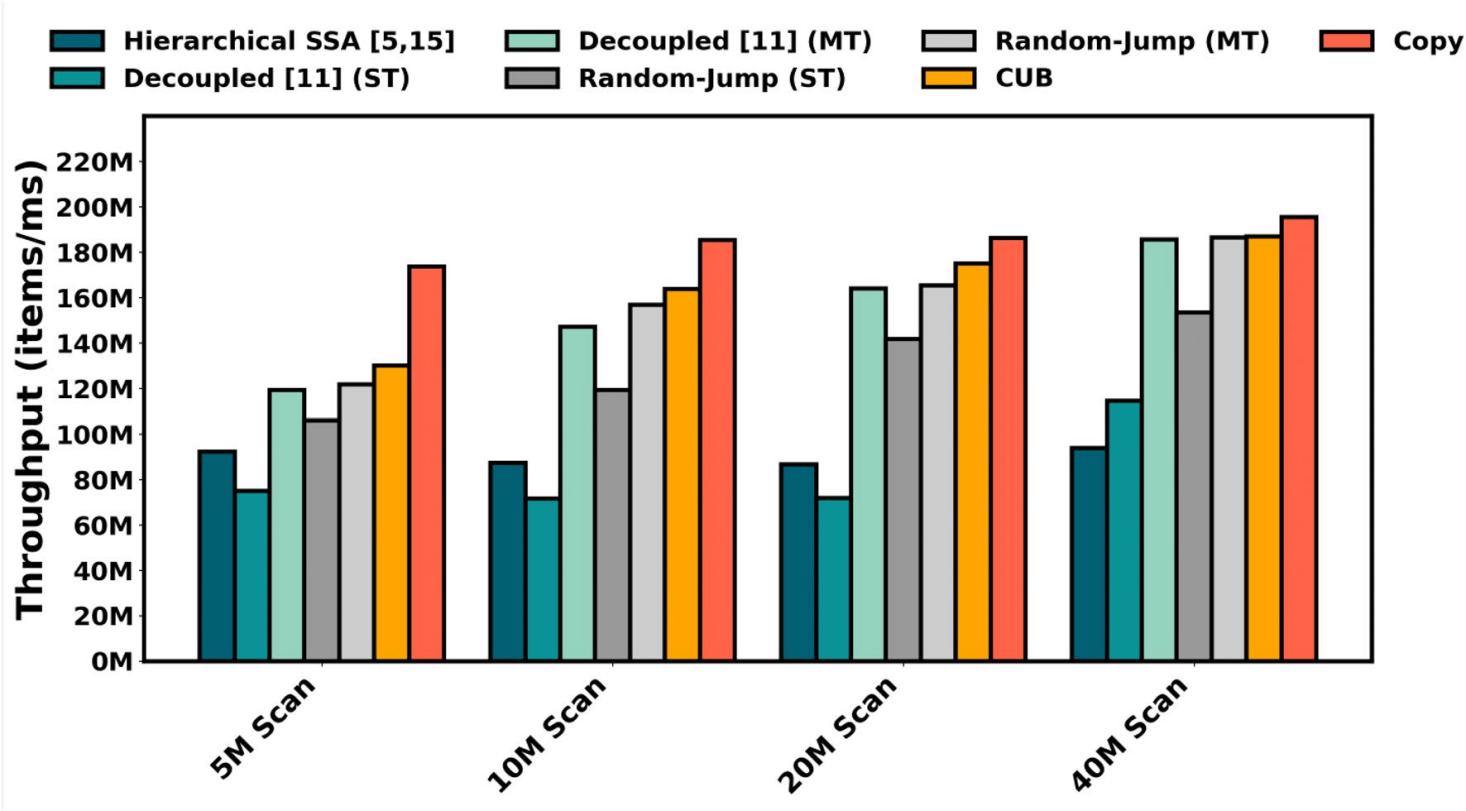
Throughput comparison of global stage scan methods on an NVIDIA A800 GPU across varying input lengths.

Figure 3 shows the throughput results for various input sequence lengths, with CUB’s DeviceScanimplementation and a naive memory-copy baseline included as reference points. Our single-threaded RandMScan implementation achieves a 59% higher throughput than the Decoupled approach from Merrill et al.^11^ and a 45% improvement over the hierarchical SSA methods of Wroblewski et al.^5^ and Dakkak et al.,^15^ reaching approximately 80% of the performance of DeviceScan. This demonstrates that our method maintains strong computational efficiency even on hardware platforms that lack conventional vector units. The multi-threaded variant of RandMScan exhibits slightly higher throughput than the multi-threaded Merrill et al. method and approaches the throughput of DeviceScan, demonstrating that RandMScan scales effectively with increased parallelism while remaining competitive with highly optimized GPU libraries.

**Table 3:** Scan execution times (ms) and speedup ratios for varying input sizes using FP16 data on the SOFA accelerator platform.

Input size	Single-pass decoupled^11^	TCU device scan^15^	MCScan^5^	Ours	Speedup
$2^{16}$	0.11	0.07	0.08	0.04	75%
$2^{18}$	0.37	0.26	0.30	0.15	73%
$2^{20}$	1.33	0.90	1.03	0.48	87%
$2^{22}$	5.41	3.69	4.31	2.04	81%
$2^{24}$	22.0	14.94	17.52	8.31	79%

To further investigate the speed-up potential of RandMScan in edge AI scenarios, we performed experiments on the SOFA accelerator^17^, a state-of-the-art edge transformer accelerator integrating two 128$\times$4 PE arrays. In our configuration, these arrays were partitioned to operate as 64 independent 4$\times$4 matrix multiplication units, allowing fine-grained parallel processing of intra-chunk computations. We benchmarked RandMScan against three representative full scan algorithms including Single-pass Decoupled^11^, TCU Device Scan^15^ and MCScan^5^ each contain both local and global stages. Table 3 summarizes the observed performance. RandMScan consistently demonstrates superior execution times, achieving speedups ranging from 73% to 87% across different input lengths. The performance benefits of RandMScan can be attributed to two primary factors: (i) during the local stage, it reduces the memory-traffic overhead associated with auxiliary matrices while achieving higher PE utilization than other matrix-based methods^5,15^; and (ii) during the global stage, where conventional multi-threading is infeasible due to the lack of SIMT units, the single-threaded Random-Jump strategy efficiently propagates prefix sums, significantly outperforming the methods proposed by Merrill et al^11^. and the hierarchical strategies used by Dakkak et al.^15^ and Wroblewski et al.^5^.

**Fig. 4:**
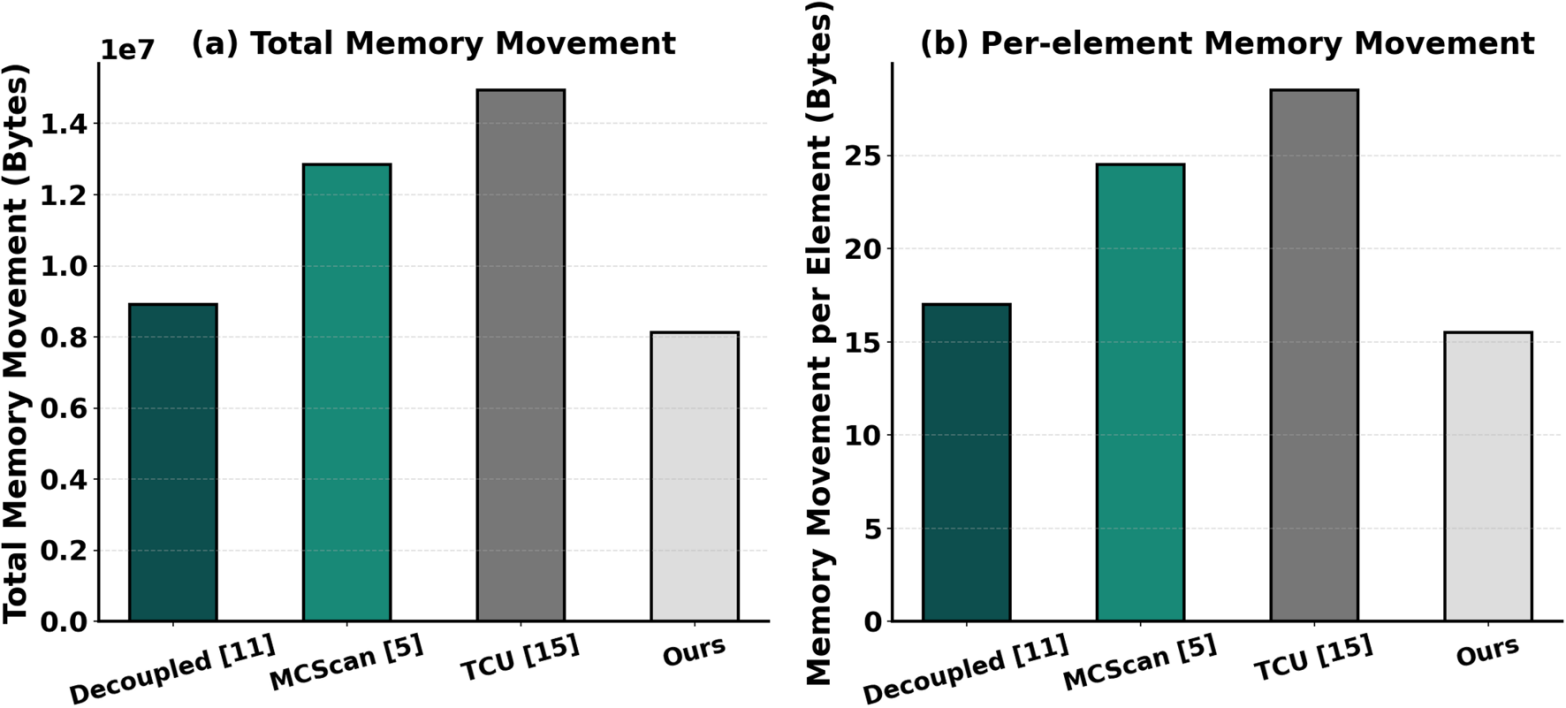
Comparison of memory movement for different scan implementations on the SOFA accelerator for an input size of $2^{20}$ elements. Both total memory transferred and per-element memory movement are shown.

To further understand the observed performance differences, we analyzed the memory movement characteristics of the different scan implementations. Fig. 4 presents the analysis for an input length of $2^{20}$ (i.e., 1M elements), comparing the total memory movement and the average bytes moved per element among different methods. It can be observed that the total memory movement of the matrix-based approaches^5,15^ and our method exhibits a similar proportional relationship to their execution time differences. In contrast, although the prefix-sum-tree-based method^11^ does not show a significantly larger memory movement than ours, its parallel prefix computation suffers from low execution efficiency on the PE array, ultimately resulting in the slowest overall performance. In addition, By reducing memory traffic and minimizing redundant auxiliary matrix transfers, RandMScan is expected to consume less energy than those baseline methods, as memory movement generally dominates the energy cost on modern deep learning accelerators^31^. Combined with its superior execution speed, this suggests that RandMScan can achieve better performance–energy trade-offs, offering both faster execution and more efficient energy utilization compared with previous scan implementations.

**Fig. 5:**
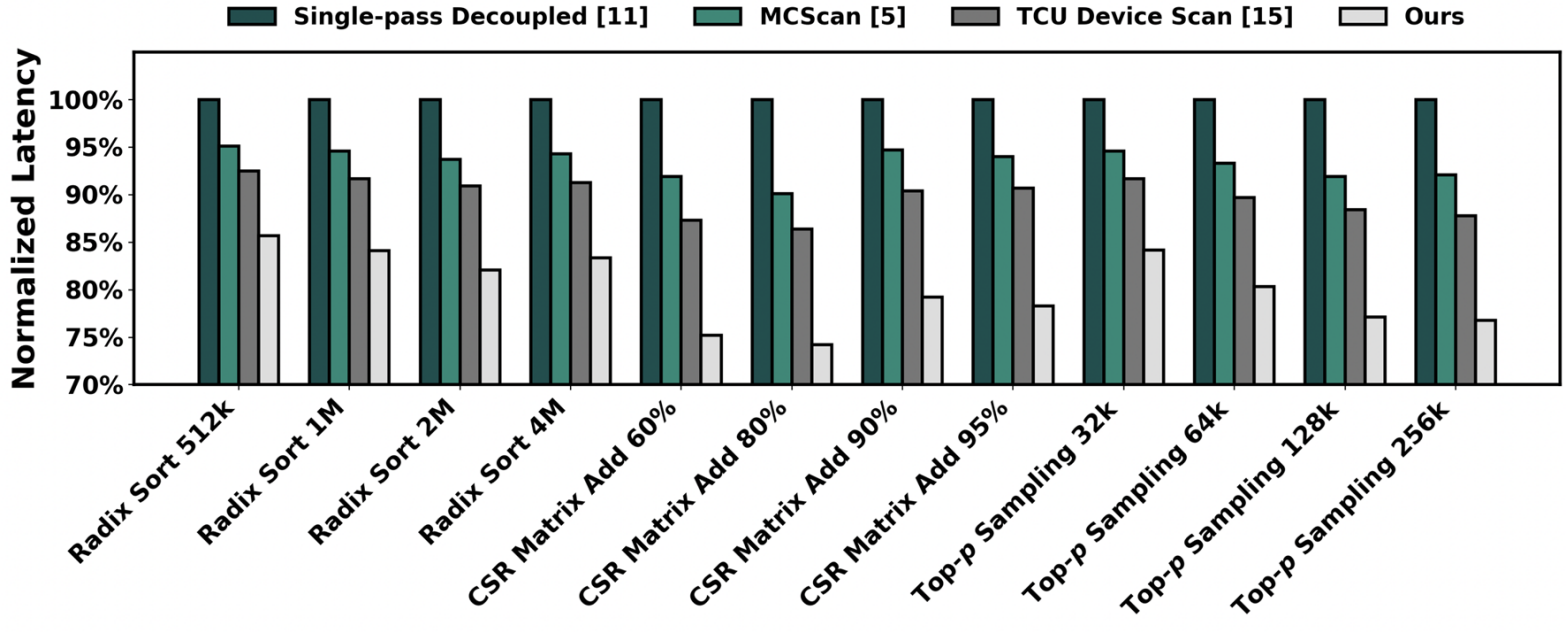
Normalized delays of several scan-based downstream tasks using different scan implementations.

We also evaluate the practical impact of RandMScan on several representative downstream tasks that rely heavily on scan operations. For radix sort and top-*p*sampling, we use commonly adopted sequence lengths of {512K, 1M, 2M, 4M} and {32K, 64 K, 128 K, 256K}, respectively. For CSR-format delta-encoding matrix addition, we benchmark matrices with 10M elements per row at sparsity levels of {60%, 80%, 90%, 95%}. In all cases, execution latencies are normalized against the baseline method reported by Merrill et al.^11^. As illustrated in Figure 5, RandMScan consistently achieves the lowest execution times across all tasks, reducing the overall latency of scan-based operations by 15% to 26% compared to existing methods. These results underscore the practical efficiency of RandMScan, demonstrating that the combined improvements in local computation and global-stage communication translate into measurable gains in end-to-end scan-dependent applications.

## Conclusion

This paper proposed a novel parallel scan implementation method for modern edge AI accelerators. First, an efficient inner-chunk matrix-based local scan algorithm was developed to minimize memory traffic and enhance computational efficiency. Then, a global scan mechanism based on a Random-Jump strategy was introduced for parallel processing of long sequences, enabling lightweight inter-chunk communication. Experimental results showed that the proposed design can achieve over 80% speedup compared with existing scan approaches on state-of-the-art accelerators, while reducing latency by 15%–26% for representative scan-based downstream tasks.

While our framework demonstrates significant performance and efficiency improvements, there remain opportunities for further enhancement. For example, the current implementation does not exploit additional hardware units available in modern accelerators, such as embedding modules, attention engines, or convolution engines, which could provide complementary acceleration for scan operations. Importantly, the proposed Random-Jump strategy remains fully effective in the current design and is compatible with future integration of such heterogeneous resources. Furthermore, the Random-Jump mechanism itself may be generalized to other parallel algorithms that involve inter-core or inter-block communication, offering a promising avenue for broader applications in large-scale parallel processing. We note that our current method assumes rectangular PE arrays, which facilitate direct mapping of square-blocked local chunks and efficient communication following GEMM-style dataflows. In non-rectangular or irregular PE arrays, such mappings may be challenging, and additional scheduling or mapping strategies would be required to maintain correctness and performance. Nevertheless, the core principle of Random-Jump accumulation remains applicable in these cases, potentially with non-trivial modifications.

## Data Availability

The datasets used and/or analysed during the current study available from the corresponding author on reasonable request.
